# Cognitive Enrichment in Piglet Rearing: An Approach to Enhance Animal Welfare and to Reduce Aggressive Behaviour

**DOI:** 10.1155/2013/389186

**Published:** 2013-10-01

**Authors:** Lilia Thays Sonoda, Michaela Fels, Sally Rauterberg, Stefano Viazzi, Gunel Ismayilova, Maciej Oczak, Claudia Bahr, Marcella Guarino, Erik Vranken, Daniel Berckmans, Jörg Hartung

**Affiliations:** ^1^Institute for Animal Hygiene, Animal Welfare and Farm Animal Behaviour, University of Veterinary Medicine Hannover, Foundation, Bünteweg 17p, 30559 Hannover, Germany; ^2^Measure, Model & Manage Bioresponses (M3-BIORES), Katholieke Universiteit Leuven, Kasteelpark Arenberg 30, 3001 Leuven, Belgium; ^3^Department of Veterinary Science and Technologies for Food Safety, Faculty of Veterinary Science, Via Celoria 10, 20133 Milano, Italy; ^4^Fancom Research, Industrieterrein 34, 5981 Panningen, The Netherlands

## Abstract

It is known that pigs raised in enriched environments express less aggressive behaviour. For this reason, a new method of cognitive environmental enrichment was experimented at the University of Veterinary Medicine Hannover, Germany. In the first phase, 78 suckling piglets were trained to learn the link between a sound given by an electronic feeder and a feed reward in the form of chocolate candies during a period of 8 days. In the second phase, the same piglets were used in resident-intruder tests to verify the potential of the feeding system to interrupt aggressive behaviour. The analysis of all training rounds revealed that piglets learned the commands during 8 days of training and the interest of the piglets increased within training days (*P* < 0.05). In the resident-intruder test, 79.5% of aggressive interactions were broken by feeder activation. In interactions where either the aggressor or the receiver reacted, a high number of fights were stopped (96.7% versus 93.1%) indicating that it was not relevant if the aggressor or the receiver responded to the feeder activation. We conclude that the electronic feeding system has the potential to be used as cognitive enrichment for piglets, being suitable for reducing aggressive behaviour in resident-intruder situations.

## 1. Introduction

In conventional pig production, pigs are usually kept without litter on slatted or at least partially slatted floors. These housing systems providing hygienic and economic benefits are often characterized by a barren environment with scant opportunities for playing or exploring and thus often not fulfilling natural behavioural needs of pigs. Furthermore, group compositions may change for several times in pig's life, and the social structure is disrupted. This procedure usually happens for the first time when piglets are weaned, a very stressful process, which can be accompanied by reduced well-being and health problems [1, 2]. After separating from the sow, unacquainted piglets of similar weight from different litters are mixed, and naturally they will fight to establish a novel social hierarchy. In order to avoid or reduce aggressive actions after weaning and mixing, several measures were already tried, such as odour masking agents, sedatives, regrouping pigs in darkness, or equipping the pen with a hiding place [3–8]. Most of these methods led only to a postponement of aggressive behaviour after mixing, without reducing it significantly. However, it was shown that environmental enrichment has the potential to reduce aggressive behaviour in groups of pigs and to avoid behavioural disorders [9]. 

A novel topic of research in this context is environmental enrichment based on cognitive challenges. Intentioned enrichment methods contain goal-directed learning behaviour and are carried out using aversive or rewarding reinforcers. These methods are expected to have immensely sustained potential to bring alternation as well as distraction and therefore to reduce boredom and abnormal behaviour, excitement, and fear behaviour [10–14]. Especially, enrichment devices which offer extrinsic reinforcement (food, social access, etc.) as a reward have proved to be effective [10]. Furthermore, cognitive tasks can lead to positive emotions, because of a controllability and predictability of the environment. Especially the successful coping with challenges induces positive experience and can improve animal welfare [11, 12, 14–17]. In addition, cognitive enrichment methods could be useful tools for an improved behavioural management having the potential to reduce unwanted behaviours and to reinforce desired behaviours [11, 12, 14]. The mentioned methods are already applied for a long time in zoos to train animals for a better handling and to bring variety in animals' daily life [18, 19]. Furthermore, cognitive enrichment achieved positive results in studies with dogs [20–22]. For farm animals, cognitive enrichment is not yet used as a common method. There are several investigations concerning learning behaviour in various farm animal species using, for example, acoustic cues to bring a desired reaction [16, 23–25]. These studies demonstrated animals' ability to adapt behaviour in order to be rewarded. Moreover, there are already simple forms of learning in farm animal housing systems where animals have to differentiate between the functions of various areas of their environment or to remember the location of food [26]. These specific behaviours can be quickly internalized, and the animals perform the required reaction automatically [12, 27]. Concerning the cognitive abilities and learning behaviour of pigs, it was shown that the foraging behaviour is a very useful issue to study links between sensory abilities, cognitive challenges, and motivational processes [14, 16, 28–31]. Pigs' auditory acuity is better than that of humans; thus, acoustic signals can serve as discriminative stimuli, or as secondary or conditioned reinforcers [27]. Furthermore, pigs seem to like sweet tastes; therefore, sweets are well suited as a reward for some challenges [27, 32].

In our study, we intended to investigate the suitability of cognitive enrichment based on a sound signal followed by a food reward for young piglets in order to reduce aggressive behaviour after weaning in conventional pig rearing systems. We used a feeder giving an acoustic signal to announce a reward in form of sweets, and suckling piglets were trained during 8 days before weaning to react on that sound. Since we worked with young animals, the reward used in the training should be attractive to suckling piglets, should be practical to be applied under farm conditions, and should be based on previous behavioural experiments in piglets [33] and grown pigs [34]; therefore, we used chocolate candies. Piglets were expected to learn to respond to the sound, to stop their current activity, and to collect the food reward. Using this method, an attempt was made to interrupt occurring fights between two piglets in resident-intruder confrontations by distracting the animals from aggressive behaviour. Thus, our main approach was to study whether cognitive enrichment applied to young suckling piglets could be able in principle to enhance management and animal welfare in later production stages by reducing excessive aggression after mixing.

## 2. Material and Methods

The experiment was divided in two parts. In the first part of the study, suckling piglets were trained to react to the activation of an electronic dog feeder (*MannersMinder*-Pet Premier, LLC). The piglets had to learn the link between a sound given by the feeder and a feed reward in form of chocolate candies during a training period of eight days. In the second part of the study, the potential of the learned behavioural responses to break or reduce aggressive behaviour between weaned piglets was tested using resident-intruder confrontations.

### 2.1. Training of Suckling Piglets

**Figure 1:**
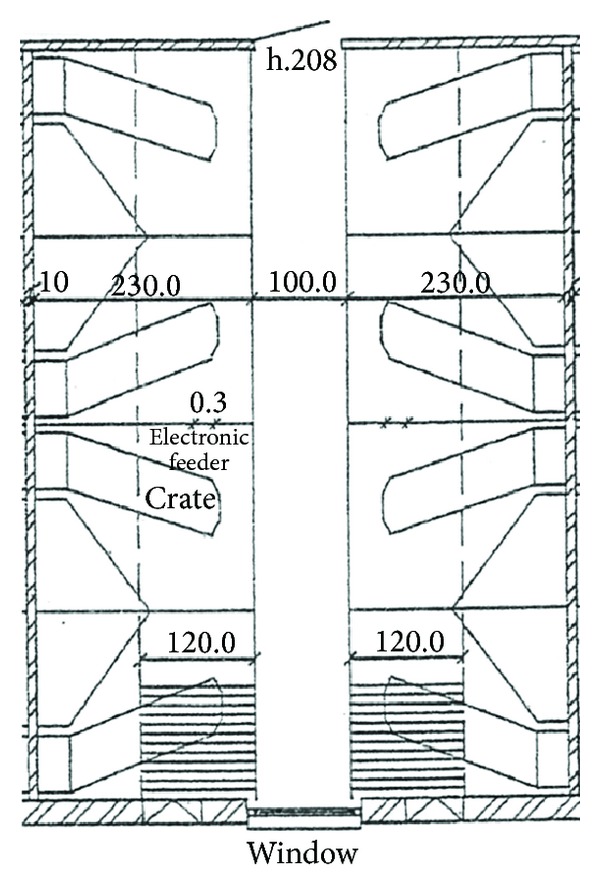
Floor plan of the farrowing pens where piglets were trained for 8 days.

Four rounds of experiment were conducted at the research farm Ruthe of the University of Veterinary Medicine Hannover, Foundation, Germany. In the research farm, a total of 90 sows were kept, and every 2 weeks five to six sows gave birth to piglets. For our study, we used the piglets of two litters per round. In total, 78 mixed sex suckling piglets of the German National Breeding Programme (BHZP) in eight entire litters with eight to 12 piglets each were studied from July 2011 to August 2012. In the beginning of the experiment, piglets were 25 days old with an average weight of 7 kg ± 1 kg. The suckling piglets were kept and trained in conventional farrowing pens (2.30 m × 2.00 m) with partially slatted floor where the sows were placed in farrowing crates as shown in Figure 1.

In the beginning of the experiment, all piglets were weighed and individually marked using standard colour stock marker.

The training consisted in the use of an electronic dog feeder that played a “beep” sound and dispensed chocolate candies after the sound. This equipment works based on a remote-controlled reward system that uses positive reinforcement for training dogs to behave appropriately at home and perform better in competitions. By rewarding desired behaviours, the system helps redirecting a dog's focus away from barking at the door, jumping on guests, or begging at the table. In our study, the equipment was used in principle to arouse piglets' curiosity and to train them to react on a sound followed by feed dispersion during a training period of 8 days. The feeder was placed on the wall of a farrowing pen with height of 0.6 m from the ground (Figure 1). The feeder was activated by a remote control, and the observer stood outside the room to limit the contact with the animals. The training started when the pigs were 25 days old and ended the day before weaning at the age of 34 days, giving a total training period of 8 days with 2 days of weekend in between. Training took one hour per day (10:00–11:00 am), and during this time the electronic feeder was activated every 10 minutes, so that training was performed 5 times per day. All the training sessions within the 8 days of training were video recorded from a top view of the pen using two cameras, a Guppy F-080C (*Allied Vision Technologies*, Germany) with a SV-03514 3.5 mm lens (*VS Technology*, Tokyo, Japan) with resolution of 1032 × 778 pixels and a Guppy GC1350 (*Allied Vision Technologies*, Germany) with a Pentax 4.8 mm lens (*Pentax Corporation*, Tokyo, Japan) and a resolution of 1360 × 1024 pixels. Both cameras were connected to a computer with LabVIEW Software (8.6, *National Instrument*, TX) that recorded synchronised videos in MJPEG format with variable frame rates between 10 and 20 images per second. The computer's processor was Intel(R) Core(TM)2 Quad CPU Q9300 @ 2.50 GHz with 6 GB of physical memory. The operating system was Microsoft Windows 7 Ultimate.

### 2.2. Resident-Intruder Test

The second part of the study was conducted in the rearing pens after weaning of trained piglets. After 8 days of training, the animals were weaned, moved to the rearing house, and mixed in groups of 12 piglets balanced by weight and sex. Per round, two groups of piglets were formed. The mean weaning weight of all piglets was 9 kg ± 1 kg, with an average weaning age of 35 days. The animals were kept without litter on totally slatted floor (0.38 m^2^ per animal, animal : feeding place ratio 1.5 : 1) separated from other piglet groups by solid pen walls. The animals had *ad libitum* access to dry food and water. One day before weaning, all piglets were individually marked again. Three days after weaning, the reaction of piglets on the use of the electronic feeder was tested during aggressive interactions, such as fighting, biting, and mounting, in a resident-intruder test.

**Figure 2:**
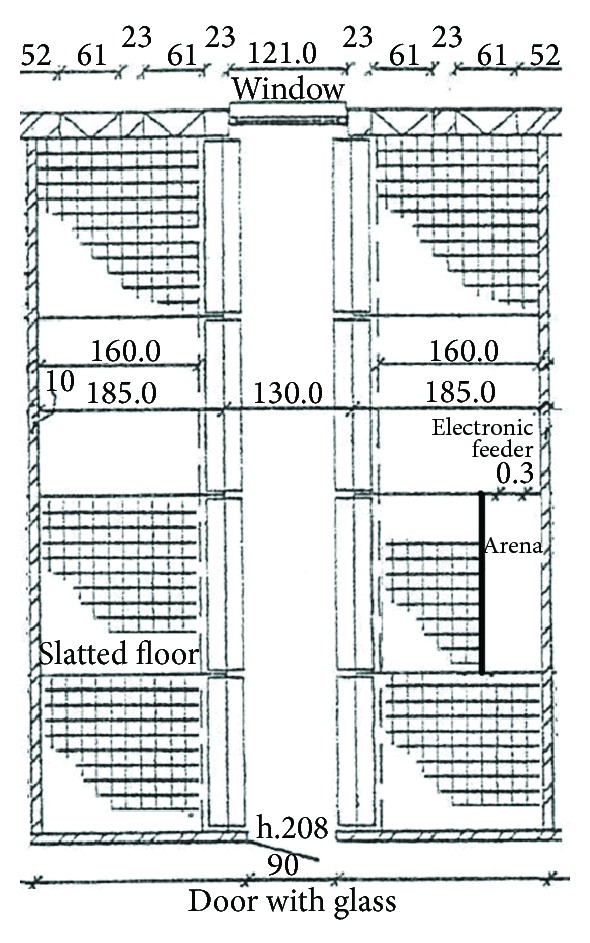
Floor plan of the weaning pens (experimental arena included) where trained piglets performed the resident-intruder test.

For this test, an arena was formed by partitioning a portion (1.0 m × 1.8 m) of the home pen (1.85 m × 1.8 m) of a group of 12 trained piglets by using a black board made of strong plastic. The electronic feeder used during the training of suckling piglets was placed on the wall of the test arena (Figure 2). 

The tests began at approximately 10:00 h in the morning, and the piglets were tested in a random order. The resident piglet was first isolated in the arena built in its home pen. The intruder piglet which came from another group of trained piglets was then collected from its home pen and placed into the test arena already containing the resident piglet. The observer stood nearby in a support at a higher level, so it was possible to see the whole area of the arena. The start of the test was defined as the time when the intruder was placed inside the arena, and it took on average 7 minutes of observation. If an attack occurred, the electronic feeder was activated in order to break the aggressive interaction. If no attack occurred within 7 minutes, the test was finished and the pairs were changed. In all other cases, the test was ended either when an escalated attack occurred that could not be broken by the activation of the electronic feeder or after 5 minutes of aggressive interactions broken by the activation of the feeder. An escalated attack was defined as a sudden rapid sequence of bites delivered by one or both pigs. Such attacks were quite distinctive, involving characteristic rapid movements of the head, and often the front legs would lift momentarily as the attacking pig's rear legs accelerated it forward. Single bites or head knocks did not lead to the end of a test, especially if they were broken by the activation of the feeder. At the end of a test, the pigs were immediately separated and moved back to their pen mates. Pairs of piglets were randomly selected. The 12 resident piglets were tested once a day with different partners for three days. All the encounters were recorded for later analysis using two cameras Guppy (described previously) placed 2.0 m central above each pen in order to have a top view perspective. Both cameras were connected to a computer with LabVIEW Software (8.6, *National Instrument*, TX) that recorded synchronised videos in MJPEG format with variable frame rates between 10 and 20 images per second. 

### 2.3. Behavioural Observations

All recorded videos were analysed using the software “Labelling Tool” [35] developed in Matlab (R2009a, The MathWorks Inc., MA, USA). The analysed behaviour was the interest of the piglets towards the training commands, and for this reason, the number of piglets around the electronic feeder was registered 2, 5, 15, 30, 45, and 60 seconds after the activation of the feeder. 

When analysing the resident-intruder test, from 82 recorded videos and a total of 268 episodes of interactions between two piglets, the observer identified which piglet started the aggressive interaction and as a consequence which piglet was the receiver. Furthermore, it was analysed if they responded to the activation of the feeder or not, if a fight could be stopped or not, and which piglet responded first or if both or none of them responded. 

### 2.4. Statistics

Statistical analysis was carried out using the statistical software package SPSS, version 20 for windows. The univariate procedure in SPSS was used to assess data for normal distribution. When analysing the training data, ANOVA analyses and post hoc tests according to student-newman-Keuls test were conducted in order to find significant differences between the tested parameters. The data of resident-intruder test were compiled in frequency tables and contingency tables, and to identify significant differences, the chi-square test was used.

## 3. Results

### 3.1. Training

Table 1 shows the results of different training days concerning the reaction of the piglets after 2, 5, 15, 30, 45, and 60 seconds of the activation of the feeder.

In the first column, the reaction of the piglets towards the training is expressed as the mean percentage of piglets around the feeder after 2 seconds of feeder activation. Although there was a significant difference between the second, the fifth, and the last day of training, the percentage of piglets around the feeder was not higher than 38% on the eighth day of training. In contrast, 5 seconds after the feeder activation, we found a higher percentage of piglets around the feeder on all training days. There was a significant difference between the first day, the second day, and the eighth day of training with the highest percentage of piglets around the feeder on the eighth day of training (63%).

After 15 seconds of the activation of the feeder, the percentage of piglets around the feeder increased reaching a maximum of 67%, and after 30 seconds of the activation of the feeder, the percentage of piglets around the feeder also increased going up to 69%. 

45 seconds after feeder activation, the percentage of piglets around the feeder was lower than the seconds before, reaching a maximum of 61% on day 3. 60 seconds after feeder activation, the number of piglets around the feeder was between 39% on day 5 and 52% on day 3, showing a decrease of interest in the feeder during the last observation. For the reaction after 60 seconds, no significant difference throughout the training days was found.

The last line of Table 1 shows the average result for the reactions of piglets in different seconds after feeder activation, independent of the training day. It can be seen that generally the number of piglets around the feeder waiting for chocolate candies increased from 2 seconds (29.56%) to 15 seconds (57%) after the feeder activation. After 15 sec, the percentage of piglets around the feeder decreased, and 60 sec after feeder activation, there were still 44% of piglets around the feeder.

### 3.2. Resident-Intruder Test

In the resident-intruder test, piglets played different roles by being aggressors or receivers during a certain confrontation.

**Figure 3:**
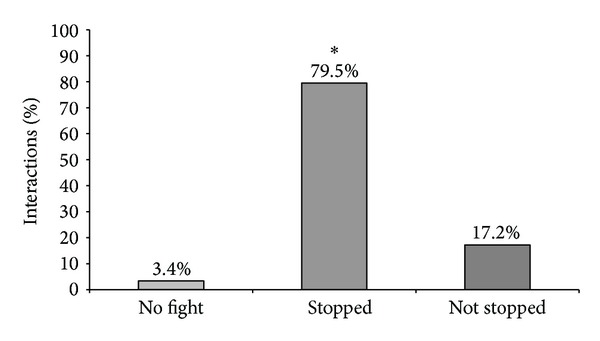
Kind of reaction (no fight, stopped, and not stopped aggressive interactions) in relation to the total percentage of interactions, *n* = 268 interactions (*P* < 0.05).

In Figure 3, the kind of reaction was divided into none, stopped, and not stopped aggressive interactions, and it was noticed that, from the total number of 268 encounters, 79.5% of the aggressive interactions could be stopped by the activation of the feeder.

**Figure 4:**
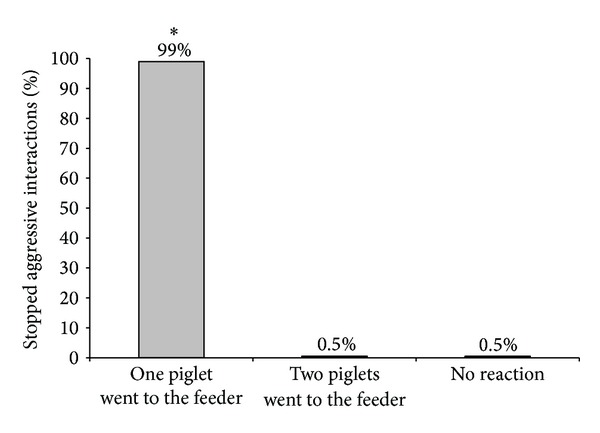
Piglets' reactions in relation to the aggressive interactions which were stopped after the activation of the feeder concerning a total of 213 stopped aggressive interactions (*P* < 0.05).

In Figure 4, it can be observed that in 99% of cases only one piglet reacted first to the feeder activation, from 213 aggressive interactions that could be stopped.

**Figure 5:**
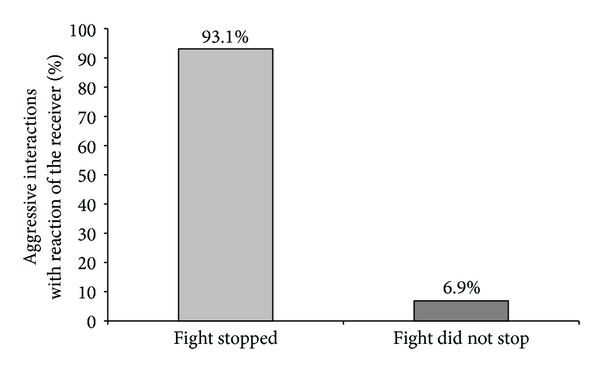
Percentage of aggressive interactions stopped and not stopped when only the receiver reacted after the activation of the feeder concerning a total of 102 stopped aggressive interactions (*P* < 0.05).

**Figure 6:**
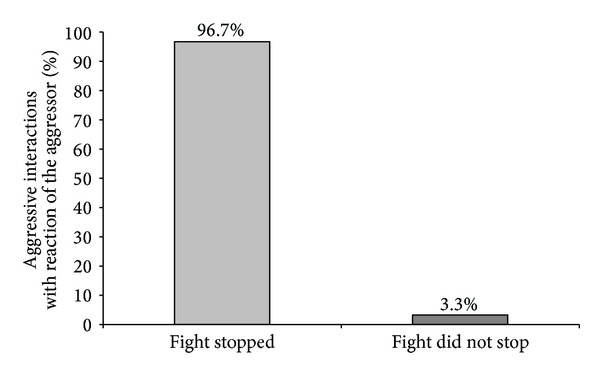
Percentage of aggressive interactions stopped and not stopped when only the aggressor reacted after the activation of the feeder concerning a total of 121 stopped aggressive interactions (*P* < 0.05).

When we verified the aggressive interactions that could be stopped when the aggressor or the receiver reacted to the activation of the feeder, we observed that when the receiver responded, around 93.1% of aggressive interactions were stopped, and when the aggressor responded, 96.7% of fights were interrupted. In 6.9% of cases, fighting did not stop although there was a reaction of the receiver to the sound signal, and in 3.3% of the cases, the aggressor reacted on feeder activation; however, piglets continued fighting (Figures 5 and 6).

**Figure 7:**
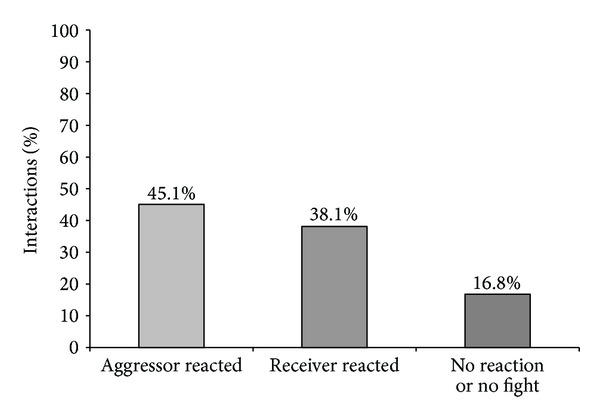
Kind of reaction (from aggressor, receiver, or no reaction or no fight) in relation to the total percentage of encounters from a total of 268 interactions (*P* < 0.05).

**Figure 8:**
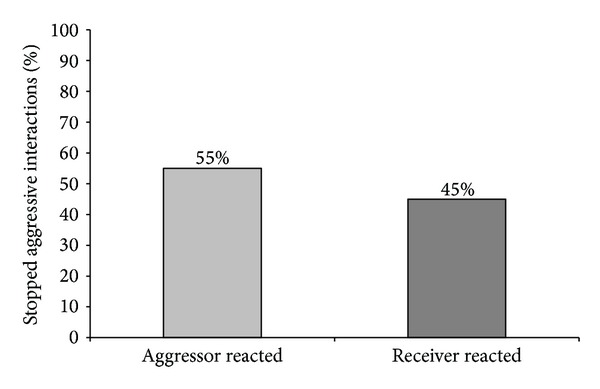
Reaction of the aggressor and the receiver in relation to the aggressive interactions which were stopped after the activation of the feeder from a total of 213 stopped aggressive interactions (*P* < 0.05).

Furthermore, the reaction of the aggressor and of the receiver towards the feeder activation and also the amount of no reaction, meaning that the piglets engaged in a fight or there was neither an aggressive interaction nor a fight, was analysed. It was observed that the aggressors tended to react slightly more than the receivers related to the total number of interactions (45% versus 38%, Figure 7) as well as those related to the total number of stopped fights (55% versus 45%, Figure 8).

## 4. Discussion

According to Puppe et al. [14], foraging behaviour of pigs is a very useful issue to study links between sensory abilities, cognitive challenges, and motivational processes. Additionally, it was already shown that pigs can differentiate an individual tone which is associated with a locally changing feeding site being able to remember a certain combination [14, 31]. The suitability of the use of an acoustic signal followed by a food reward for conditioning of piglets was also confirmed in our study. In contrast to other studies, where older pigs were trained, we used young suckling piglets, which were still in the farrowing crates with their mother sows, and we showed that even at this young age a food rewarded training was possible.

For the training, the piglets in our study took eight days to present increasing interest. Similar results obtained by Laughlin and Mendl [36] confirm the need for at least 10 days of training for 16 Large White x Landrace male pigs which were 10–12 weeks old to remember some training commands and to display working behaviour related to these commands with a high successful rate. When analysing the different training days, we found that specifically from the third day of training, the piglets showed significant improvement regarding their response towards the training commands, and it continued until the end of the training, on the eighth day. Usually, pigs need a certain time to get accustomed to a novel object or a change in their routine, so it is plausible to believe that, after the period of habituation, the piglets relaxed and learned quicker, presenting themselves in a higher percentage around the feeder. When Mendl et al. [37] trained eight Large White Landrace male pigs of 48 kg for performing a foraging task, they reported that cases of defecation, a possible indicator of fearfulness, were observed for maximum of 2-3 days after the training started. Nevertheless, there were never 100% of piglets of a litter around the feeder at the same time. As the piglets in our experiment were 2.5 weeks old when training started, they were still initiating with rooting behaviour and not very familiar with solid food. For this reason, the sow represented the most important food resource at that moment, having a bigger influence on the piglets' behaviour than dry feed itself [38, 39]. It was already shown that even though creep feed was provided *ad libitum*, piglets relied mainly on the sow's milk, which provides them not only nutrients but also immunoglobulins, bioactive proteins, and peptides stimulating the development of the stomach and small intestine [40, 41]. By the fact that the piglets were trained in the presence of the sow, it can possibly be explained why the percentage of the piglets around the feeder was not higher than 69% in total. 

Although the training was done in the morning when piglets were more active, 2 seconds after the sound was played, that is, at the moment of the food release, the percentage of piglets around the feeder was not higher than 38%. One possible reason for this result can be simply the amount of time that the piglets needed to approach the feeder because, on the other hand, after 5, 15, and 30 seconds after the activation of the feeder, the percentage of piglets around the feeder was much higher with 63%, 67%, and 69%, respectively. Another possibility can be the amount of time which was necessary to make the connection between the sound and process the information. When Puppe et al. [14] evaluated the learning behaviour of 112 castrated German Landrace male pigs starting at the age of 7 weeks, they found that the animals, after 20 weeks of training, had a success rate of 80% and the time spent from an acoustical signal to the release of food by a “call-feeding-station” took approximately 15 seconds. Zebunke et al. [16] worked with 24 German Landrace pigs at the age of 16 weeks and reported the same time of 15 seconds from the time of the call until the pigs approached the feed, dispensed by an automatic feeding station, even after 7 weeks of training. 

In addition, Croney [42] found that pigs performed better on discrimination learning tasks when olfactory rather than visual stimuli were used. In this sense, a farm environment can be disturbing when taking into account the hearing of the animals, by the fact that equipment and other animals are emitting sounds at the same time. 

After 45 and 60 seconds of feeder activation, the number of piglets around the feeder decreased, which can be explained by the fact that chocolate candies were lacking and piglets lost interest at that latest moment of observation. Also, Held et al. [28, 30] and Croney et al. [29] found that, generally, pigs are highly motivated if food is introduced as enrichment; however, this motivation can vary when the level of competition is high and a constant amount of food is not maintained [14]. This could also explain why never 100% of piglets were observed around the feeder. The food release caused also competition between the piglets with the stronger piglets having privileged access to food. Weaker piglets could probably only reach the food, when most chocolate candies were already eaten by stronger piglets. For technical reasons and because of lack of space, we could not offer a trough where all piglets were able to eat at the same time without disturbing each other. Nevertheless, good training results were obtained, which were confirmed by a high efficiency of the feeder in interrupting aggressive interactions during the resident-intruder test. We showed that even under field conditions with limited space allowance in a conventional farrowing crate, it is possible to offer cognitive enrichment and to train young piglets to react on certain stimuli.

To induce aggressive behaviour among two piglets and to act on individual animals, the method of resident-intruder confrontation was chosen permitting a good overview and control of two interacting animals. Using a resident-intruder situation, the training commands were proven to have positive effects during aggressive interactions since 79.5% of all fights could be stopped by the activation of the electronic feeder, showing that cognitive enrichment is generally suitable to influence aggressive behaviour in piglets. From earlier studies, it is known that aggressive behaviour can be affected in principle by environmental enrichment. Schaefer et al. [9] showed the effectiveness of environmental enrichment in reducing total aggression and the improvement of animal growth by testing different materials such as car tires, sugar-mineral blocks, or teeter-totter. O'Connell and Beattie [43] also verified significant less fights between animals raised in environments provided with straw. Olsson et al. [44], who tried a different approach in their experimental design by providing sand to piglets in the farrowing unit in order to reduce aggressive interactions during resident-intruder confrontations, found that piglets raised in environments with no bedding materials inflicted more wounds on each other during dyadic confrontations. Furthermore, the establishment of social dominance relationships was impaired in these animals compared to pigs from enriched rearing conditions. However, all of these methods of environmental enrichment have something in common which is that they were not using cognitive challenges and thus are different from our own study which is, to our best knowledge, the first study investigating the efficiency of cognitive enrichment to influence aggressive behaviour in pigs.

Regarding the number of piglets going to the feeder after the activation, we found that in 99% of cases only one piglet responded immediately to the sound so that fighting stopped. This piglet could be the aggressor or the receiver, since we found no significant difference between the percentages of reaction of aggressor or receiver when an aggressive interaction was stopped by feeder activation (55% versus 45%). Also regarding the total number of encounters, there was no significant difference between the number of reactions of aggressor or receiver (45% versus 38%). We found only a tendency for aggressors to react slightly better to the activation of the feeder in a resident-intruder situation than the receivers. However, if the aggressor or the receiver reacted was not important for the effectiveness of feeder activation on the interruption of fights. We saw similar high values of stopped fights when the aggressor or the receiver reacted (96.7% versus 93.1% of fights were stopped). In principle, our hypothesis was that the most aggressive pigs would probably be the best to react to the activation of the feeder, by the natural motivation of pigs for competing for resources [45, 46], in our case, the chocolate candies. However, from our results, we conclude that it is not essential to influence the piglet that starts fighting in order to stop the aggressive behavior; it can also be useful to act on the piglet that is attacked to interrupt a fight. Thus, it was shown that the role that each piglet performed, as aggressor or as receiver, did not influence its response to the feeder activation, but it was also demonstrated that cognitive enrichment could have a high potential to interrupt fighting between two piglets. Regarding aggressiveness in resident-intruder situations, several studies were already carried out; however, no information was found concerning the reactions of piglets on different actuators during fighting. When Erhard and Mendl [47] and D'Eath [48] tested piglets in resident-intruder situations, they could not find any connection between being the resident or intruder during a confrontation and its aggressive score, as well as no connection regarding sex, age, and body weight. From these results, it can be concluded that the performed behaviour in a resident-intruder confrontation is mainly caused by individual differences in aggressiveness expressed by different piglets. Individual differences in aggressiveness were already reported in other studies searching for behavioural parameters related to aggression. Forkman et al. [49] and D'Eath and Burn [50], who analysed the performance of piglets in a Backtest, could not find a connection between being high or low resistant and individual aggressiveness. However, when Erhard et al. [51] and Bolhuis et al. [52] evaluated high or low resistant pigs after mixing, high resistant pigs were shown to be more aggressive towards their penmates. Ruis et al. [53] observed the same relation while analysing 76 low and high resistant female pigs. The high resistant gilts showed more aggression in group-feeding competition tests. Spake et al. [54] tested whether there was a correlation between the Backtest performance as an indicator of aggressive behaviour in a resident-intruder test and the connection between fear/curiosity in response to a novel object test. Neither the Backtest nor the novel object test performances were behavioural indicators for aggressiveness in a resident-intruder test. Individual differences in coping style towards a certain situation may also explain why in our study no relationship between being aggressor or receiver, and the reactions to feeder activation were found. 

## 5. Conclusion

In this study, it was shown that cognitive enrichment based on connecting a sound signal to a food reward applied in young suckling piglets can be used to influence aggressive behaviour in resident-intruder confrontations after weaning. We showed that it is possible to train piglets under practical conditions in their farrowing crates to react on a sound signal which can be used after weaning, to influence their behaviour, particularly during aggressive interactions. Thus, we conclude that cognitive enrichment is generally suitable for pigs even at a very young age having the potential to affect behaviour in later production stages. Further research is needed to investigate if this kind of enrichment can also be used to interrupt aggressive interactions of pigs kept in groups, and thus, could attain practical relevance.

## Figures and Tables

**Table 1:** Percentage of piglets around the feeder after 2, 5, 15, 30, 45, and 60 seconds of the activation of the feeder on different training days. Different letters indicate significant differences (*P* < 0.05).

Reaction in:	2 sec (%)	5 sec (%)	15 sec (%)	30 sec (%)	45 sec (%)	60 sec (%)
Day 1	26.05	34.68^a^	43.40^a^	36.69^a^	43.24^a^	42.06
Day 2	18.76^a^	30.38^a^	44.23^a^	48.12	47.10	44.81
Day 3	28.58	54.83^b^	67.63^b^	69.19^b^	61.87^b^	52.24
Day 4	33.89	50.45	56.54	56.34	52.21	43.09
Day 5	22.47^a^	45.32^b^	52.82	50.26	45.80	39.82
Day 6	31.80	55.92	56.99	54.54	50.34	43.76
Day 7	28.10	55.58	61.38^b^	59.70	58.59	48.78
Day 8	38.24^b^	63.17^b^	63.25^b^	59.20^b^	51.50	43.80

All days (means)	29.56	51.21	57.00	55.17	51.51	44.44

## References

[1] Achilles W, Bicker M, Haidn B (2005). *Stallbaulösungen für die Ferkelaufzucht*.

[2] Dybkjær L (1992). The identification of behavioural indicators of “stress” in early weaned piglets. *Applied Animal Behaviour Science*.

[3] Petherick JC, Blackshaw JK (1987). A review of the factors influencing the aggressive and agonistic behaviour of the domestic pig. *Australian Journal of Experimental Agriculture*.

[4] Arey DS, Edwards SA (1998). Factors influencing aggression between sows after mixing and the consequences for welfare and production. *Livestock Production Science*.

[5] Olesen LS, Nygaard CM, Friend TH (1996). Effect of partitioning pens on aggressive behavior of pigs regrouped at weaning. *Applied Animal Behaviour Science*.

[6] Francis DA, Christison GI, Cymbaluk NF (1996). Uniform or heterogeneous weight groups as factors in mixing weanling pigs. *Canadian Journal of Animal Science*.

[7] Tan SSL, Shackleton DM (1990). Effects of mixing unfamiliar individuals and of azaperone on the social behaviour of finishing pigs. *Applied Animal Behaviour Science*.

[8] Amstutz M, Bennett-Wimbush K, Meek T, Courtney S (2005). Effects of Acclimate on the frequency and duration of aggressive sequence and growth performance in co-mingled, weaned pigs. *Livestock Production Science*.

[9] Schaefer AL, Salomons MO, Tong AKW, Sather AP, Lepage P (1990). The effect of environment enrichment on aggression in newly weaned pigs. *Applied Animal Behaviour Science*.

[10] Tarou LR, Bashaw MJ (2007). Maximizing the effectiveness of environmental enrichment: suggestions from the experimental analysis of behavior. *Applied Animal Behaviour Science*.

[11] Manteuffel G, Langbein J, Puppe B (2009). From operant learning to cognitive enrichment in farm animal housing: bases and applicability. *Animal Welfare*.

[12] Manteuffel G, Langbein J, Puppe B (2009). Increasing farm animal welfare by positively motivated instrumental behaviour. *Applied Animal Behaviour Science*.

[13] Meehan CL, Mench JA (2007). The challenge of challenge: can problem solving opportunities enhance animal welfare?. *Applied Animal Behaviour Science*.

[14] Puppe B, Ernst K, Schön PC, Manteuffel G (2007). Cognitive enrichment affects behavioural reactivity in domestic pigs. *Applied Animal Behaviour Science*.

[15] Meyer S, Puppe B, Langbein J (2010). Kognitive Umweltanreicherung bei Zoo- und Nutztieren—Implikationen für Verhalten und Wohlbefinden der Tiere. *Berliner und Münchener Tierärztliche Wochenschrift*.

[16] Zebunke M, Langbein J, Manteuffel G, Puppe B (2011). Autonomic reactions indicating positive affect during acoustic reward learning in domestic pigs. *Animal Behaviour*.

[17] Dantzer R (2002). Can farm animal welfare be understood without taking into account the issues of emotion and cognition?. *Animal Science*.

[18] Reinhardt V (2003). Working with rather than against macaques during blood collection. *Journal of Applied Animal Welfare Science*.

[19] Schapiro SJ, Bloomsmith MA, Laule GE (2003). Positive reinforcement training as a technique to alter nonhuman primate behavior: quantitative assessments of effectiveness. *Journal of Applied Animal Welfare Science*.

[20] Schipper LL, Vinke CM, Schilder MBH, Spruijt BM (2008). The effect of feeding enrichment toys on the behaviour of kennelled dogs (Canis familiaris). *Applied Animal Behaviour Science*.

[21] Pongrácz P, Miklósi Á, Kubinyi E, Topál J, Csányi V (2003). Interaction between individual experience and social learning in dogs. *Animal Behaviour*.

[22] Pongrácz P, Miklósi Á, Kubinyi E, Gurobi K, Topál J, Csányi V (2001). Social learning in dogs: the effect of a human demonstrator on the performance of dogs in a detour task. *Animal Behaviour*.

[23] Wredle E, Munksgaard L, Spörndly E (2006). Training cows to approach the milking unit in response to acoustic signals in an automatic milking system during the grazing season. *Applied Animal Behaviour Science*.

[24] Ernst K, Puppe B, Schön PC, Manteuffel G (2005). A complex automatic feeding system for pigs aimed to induce successful behavioural coping by cognitive adaptation. *Applied Animal Behaviour Science*.

[25] Kiley-Worthington M, Savage P (1978). Learning in dairy cattle using a device for economical management of behaviour. *Applied Animal Ethology*.

[26] Wechsler B, Lea SEG (2007). Adaptation by learning: its significance for farm animal husbandry. *Applied Animal Behaviour Science*.

[27] Gieling ET, Nordquist RE, van der Staay FJ (2011). Assessing learning and memory in pigs. *Animal Cognition*.

[28] Held S, Baumgartner J, Kilbride A, Byrne RW, Mendl M (2005). Foraging behaviour in domestic pigs (Sus scrofa): remembering and prioritizing food sites of different value. *Animal Cognition*.

[29] Croney CC, Adams KM, Washington CG, Stricklin WR (2003). A note on visual, olfactory and spatial cue use in foraging behavior of pigs: indirectly assessing cognitive abilities. *Applied Animal Behaviour Science*.

[30] Held S, Mendl M, Devereux C, Byrne RW (2000). Social tactics of pigs in a competitive foraging task: the ‘informed forager’ paradigm. *Animal Behaviour*.

[31] Laughlin K, Mendl M (2000). Pigs shift too: foraging strategies and spatial memory in the domestic pig. *Animal Behaviour*.

[32] Kennedy JM, Baldwin BA (1972). Taste preferences in pigs for nutritive and non-nutritive sweet solutions. *Animal Behaviour*.

[33] de Jonge FH, Tilly S-L, Baars AM, Spruijt BM (2008). On the rewarding nature of appetitive feeding behaviour in pigs (Sus scrofa): do domesticated pigs contrafreeload?. *Applied Animal Behaviour Science*.

[34] Arts JWM, van der Staay FJ, Ekkel ED (2009). Working and reference memory of pigs in the spatial holeboard discrimination task. *Behavioural Brain Research*.

[35] Viazzi S, Ismayilova G, Sonoda L Labelling of video images: the first step to develop an automatic monitoring tool of pig aggression.

[36] Laughlin K, Mendl M (2004). Costs of acquiring and forgetting information affect spatial memory and its susceptibility to interference. *Animal Behaviour*.

[37] Mendl M, Laughlin K, Hitchcock D (1997). Pigs in space: spatial memory and its susceptibility to interference. *Animal Behaviour*.

[38] Jensen P, Redbo I (1987). Behaviour during nest leaving in free-ranging domestic pigs. *Applied Animal Behaviour Science*.

[39] Pluske JR, Le Dividich J, Verstegen MWA (2003). *Weaning the Pig: Concepts and Consequences*.

[40] Zabielski R (1998). Regulatory peptides in milk, food, and in the gastrointestinal lumen of young animals and children. *Journal of Animal and Feed Sciences*.

[41] Zabielski R, Gajewski Z, Valverde Piedra JL (2007). The perinatal development of the gastrointestinal tract in piglets can be modified by supplementation of sow diet with bioactive substances. *Livestock Science*.

[42] Croney C (1999). *Cognitive abilities of domestic pigs (Sus scrofa) [Dissertation]*.

[43] O’Connell NE, Beattie VE (1999). Influence of environmental enrichment on aggressive behaviour and dominance relationships in growing pigs. *Animal Welfare*.

[44] Olsson IAS, de Jonge FH, Schuurman T, Helmond FA (1999). Poor rearing conditions and social stress in pigs: repeated social challenge and the effect on behavioural and physiological responses to stressors. *Behavioural Processes*.

[45] Andersen IL, Andenæs H, Bøe KE, Jensen P, Bakken M (2000). The effects of weight asymmetry and resource distribution on aggression in groups of unacquainted pigs. *Applied Animal Behaviour Science*.

[46] Keeling L, Gonyou H (2001). *Social Behaviour in Farm Animals*.

[47] Erhard HW, Mendl M (1997). Measuring aggressiveness in growing pigs in a resident-intruder situation. *Applied Animal Behaviour Science*.

[48] D’Eath RB (2002). Individual aggressiveness measured in a resident-intruder test predicts the persistence of aggressive behaviour and weight gain of young pigs after mixing. *Applied Animal Behaviour Science*.

[49] Forkman B, Furuhaug IL, Jensen P (1995). Personality, coping patterns, and aggression in piglets. *Applied Animal Behaviour Science*.

[50] D’Eath RB, Burn CC (2002). Individual differences in behaviour: a test of ‘coping style’ does not predict resident-intruder aggressiveness in pigs. *Behaviour*.

[51] Erhard HW, Mendl M, Ashley DD (1997). Individual aggressiveness of pigs can be measured and used to reduce aggression after mixing. *Applied Animal Behaviour Science*.

[52] Bolhuis JE, Schouten WGP, Schrama JW, Wiegant VM (2005). Individual coping characteristics, aggressiveness and fighting strategies in pigs. *Animal Behaviour*.

[53] Ruis MAW, te Brake JHA, van de Burgwal JA, de Jong IC, Blokhuis HJ, Koolhaas JM (2000). Personalities in female domesticated pigs: behavioural and physiological indications. *Applied Animal Behaviour Science*.

[54] Spake JR, Gray KA, Cassady JP (2012). Relationship between backtest and coping styles in pigs. *Applied Animal Behaviour Science*.

